# Effect of Gestational Diabetes on Postpartum Depression-like Behavior in Rats and Its Mechanism

**DOI:** 10.3390/nu14061229

**Published:** 2022-03-14

**Authors:** Runlong Zhao, Yalin Zhou, Hanxu Shi, Wanyun Ye, Ying Lyu, Zhang Wen, Rui Li, Yajun Xu

**Affiliations:** 1 Department of Nutrition and Food Hygiene, School of Public Health, Peking University, No. 38 Xueyuan Road, Beijing 100083, China; 1510306204@pku.edu.cn (R.Z.); zylyingyang@163.com (Y.Z.); shihanxu@pku.edu.cn (H.S.); yewanyun_vera@bjmu.edu.cn (W.Y.); lybjmu@126.com (Y.L.); 1710306240@pku.edu.cn (Z.W.); lr15321657608@163.com (R.L.); 2 PKUHSC—China Feihe Joint Research Institute of Nutrition and Healthy Lifespan Development, No. 38 Xueyuan Road, Beijing 100083, China; 3 Beijing Key Laboratory of Toxicological Research and Risk Assessment for Food Safety, Peking University, No. 38 Xueyuan Road, Beijing 100083, China

**Keywords:** gestational diabetes mellitus, postpartum depression, tryptophan metabolism, serotonin, kynurenine, gut microbiota

## Abstract

Recent studies have reported a strong association between gestational diabetes mellitus (GDM) and postpartum depression (PPD), but little is known about the underlying physiological mechanism. In this study, a GDM rat model was used to evaluate the direct effect of GDM on PPD and to explore the mechanism. After parturition, the GDM dams were divided into two groups: blood glucose not recovered group (GH group) and blood glucose recovered group (GL group). Fasting plasma glucose (FPG), cortisol (COR) and serotonin (5-hydroxytryptamine, 5-HT) metabolism were continuously monitored during the lactation period, until postnatal day 21. PPD was evaluated by behavioral tests. At the endpoint, the expression of the key enzymes of Trp metabolic pathway in colon and brain tissues was analyzed by immunohistochemistry and western blot. The microbe composition of colonic contents was determined by 16S rDNA gene sequencing. The results showed that GDM induced postpartum depression-like behavior in rats. The HPA axis hormone did not show the typical stress state of depression, but the level of 5-HT decreased significantly in serum, prefrontal cortex and hippocampus, and the Kyn/Trp ratio increased significantly in serum and prefrontal cortex, implying the switch of the tryptophan (Trp) metabolism from the 5-HT pathway to the kynurenine (Kyn) pathway. The expression of Indoleamine 2,3-dioxygenase (IDO), a key rate-limiting enzyme in Kyn metabolism, was up-regulated in the colon and brain, which was an important reason for this switch. This switch was accelerated by a decrease in the expression of tryptophan hydroxylase (TPH), a key enzyme of the 5-HT production pathway, in the colon. GDM dams displayed significant changes in gut microbiome profiles, which were correlated with depression. The ratio of *Firmicutes* to *Bacteroidetes* decreased. *Lactobacillus* and *Bacteroides* were negatively correlated with 5-HT level and positively correlated with Kyn level, whereas *Clostridium* XlVa and *Ruminococcus* were positively correlated with 5-HT level. These results suggest that GDM disrupts both the Trp pathway and the composition of the gut microbiota, which provide a putative physiological basis for PPD.

## 1. Introduction

Postpartum depression (PPD) is a common type of puerperal mental disorder characterized by persistent and severe depression and a range of symptoms. Symptoms usually appear within 2 weeks after birth. It has been reported that one in seven new mothers is affected by PPD and that the prevalence of depression within one year after delivery is 21.9% [1]. PPD directly affects the mother’s mental health and social adaptability and also interferes with her ability to adequately breastfeed and care for her child [2]. Under this circumstance, the infants are at risk of suffering from delayed, even impaired, physical and mental development. Some women with postpartum major depression may experience suicidal ideation or obsessive thoughts of harming their infants [3].The mechanism of PPD has not been fully elucidated; however, it is generally accepted that both physiological and psychosocial factors play an important role.

**Figure 1:**
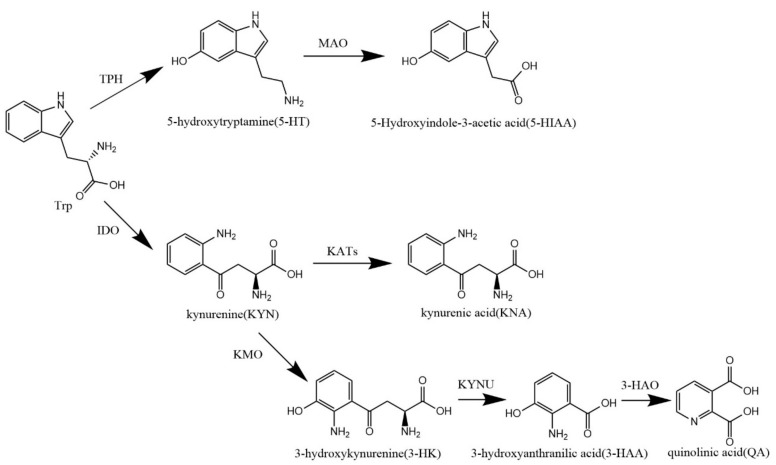
Trp metabolic pathway.

One of the known PPD mechanisms is related to the hypothalamic-pituitary-adrenal (HPA) axis signaling pathway. Researchers have demonstrated in mice that postpartum HPA axis dysfunction is sufficient to induce postpartum depression, with maternal increased HPA axis excitability and glucocorticoid secretion [4,5,6]. Changes in neurotransmitters, particularly serotonin (5-hydroxytryptamine, 5-HT), which induces happy emotions, are the pathophysiology basis of mood disorders [7]. 5-HT is mainly metabolized by tryptophan (Trp) and distributed in the intestinal mucosa and brain, with some circulation in peripheral blood. When tryptophan metabolism is disordered, 5-HT synthesis is decreased, and kynurenine (Kyn) synthesis is increased (Figure 1). However, Kyn and its downstream metabolites have been shown to be associated with depression and neuronal damage [7,8].

In recent years, there has been a growing body of research on the effects of gut microbes on mood through the gut–brain axis; high levels of *Faecalibacterium* and *Coprococcus* were associated with good mental health, whereas high levels of *flavobacterium* were associated with poor mental health. The composition of the gut microbiota of patients with depression is different from that of normal people, which may be related to the ability of microorganism to produce neuroactive substances [9,10].

A large number of studies have confirmed that patients with gestational diabetes mellitus (GDM) have a higher PPD risk than women with normal blood glucose levels during pregnancy [11,12,13]. Although several studies have focused on the relationship between GDM and PPD, most have found it difficult to determine the effect of plasma glucose on the development of PPD in patients with GDM; since people are in a complex social environment, it is difficult to completely remove the social and environmental factors and independently study the effect of the physiological factors of GDM on PPD. As a result, most studies attribute the association between GDM and PPD solely to psychosocial effects, that is, the social and psychological stress caused by the disease [14,15]. Whether GDM itself has direct effect on the occurrence of PPD and the mechanism are seldom reported.

In this study, the direct effect of GDM on the development of PPD was studied with a GDM rat model, excluding confounding by social and psychological factors, in order to provide a scientific basis for PPD early prevention and intervention.

## 2. Materials and Methods

### 2.1. Animals

Sprague-Dawley (SD) rats of specific-pathogen-free (SPF) degree were used throughout the study. Female and male rats were obtained from the Department of Laboratory Animal Science of Peking University (Beijing, China, SCXK-2012-0015) and kept in a barrier environment with a temperature of 22 ± 2 °C, humidity of 50–60%, and 12 h/12 h light/dark cycle. Rats were provided distilled water ad libitum. The experiment was approved by the ethics committee of Peking University (No. LA2021107).

### 2.2. Establishing the GDM Model

The combination of high-fat diet (HFD) with an intraperitoneal injection of 30 mg/kg streptozotocin (STZ) was adopted to establish the GDM rat model [16]. Four-week-old female rats were randomly grouped into the control group (CON) and GDM group after adaptive feeding for one week. The rats in the CON group were fed with basic chow, and the rats in the GDM group were fed HFD (HFD formula: lard 10%, sucrose 15%, egg yolk powder 15%, casein 5%, cholesterol 1.2%, sodium cholate 0.2%, calcium bicarbonate 0.6%, stone powder 0.4%, rat maintenance feed 52.6%) for 8 weeks. After that, following fasting for 12 h, tail vein blood was collected, and the fasting plasma glucose (FPG) was measured using a rapid blood glucose detector (OneTouch Ultraeasy, LifeScan Inc., Milpitas, CA, USA). Rats with FPG > 6.67 mmol/L were removed from the study due to violating the definition of GDM (carbohydrate intolerance of variable severity with onset or first recognition during pregnancy, not before gestation). Eligible female rats were mated with healthy male rats overnight at 1:1. The next morning, vaginal smears were observed under a microscope to judge the successful pregnancy. The presence of sperm in the vaginal smear was considered gestation day 0 (GD0). On GD5, after fasting overnight, pregnant rats in the GDM group were intraperitoneally injected with 30 mg/kg 1% STZ (STZ dissolved in 0.1 mol/L sodium citrate solution, pH = 4.2), and the rats in CON group were intraperitoneally injected with 0.1 mol/L sodium citrate solution. Seventy-two hours after injection, pregnant rats with FPG > 6.67 mmol/L were considered a successful model of GDM [17].

### 2.3. Experimental Groups

All the dams were allowed to give birth naturally and breastfeed their offspring. According to the FPG levels measured on day 4 postpartum, the dams of the GDM group were further divided into two groups, the postpartum blood glucose not recovered group (GH group) and recovered group (GL group). Those dams with FPG levels exceeding two standard deviations of the mean FPG of the CON group were classified as the GH group, and the remain dams in the original GDM group were classified as the GL group.

### 2.4. Behavioral Testing

After delivery, all rats were fed with basic diet, and a series of behavioral tests were carried out.

#### 2.4.1. Open Field Test

The open field test (OFT) was used to assess the exploratory and anxious behavior of rats in a new environment [18]. The open box was made of opaque black organic plastic (100 × 100 × 50 cm) and divided into 25 identical squares (20 × 20 cm) with white stripes. The square area in the middle of the box was the central region (60 × 60 cm), which included 9 squares. This test was performed in a quiet and dark (visibility was 5 m) room. A single rat was placed in the center of the area and was let for exploration for 3 min. After each test, the box was swabbed with 75% alcohol. The number of squares crossed by each rat (with three paws crossing the line), the number of standing times (with two front paws off the ground) and the time spent in the central region (with all four paws in the central region) were recorded. The OFT was carried out 3 times, which were on PND4, PND11 and PND18.

#### 2.4.2. Elevated Plus Maze Test

The elevated plus maze (EPM) test is one of the most widely used models for evaluating rat depression-like behavior, taking advantage of animals’ exploratory nature to novel environments and their fear of overhanging arms to form a conflict to examine animal depression [19]. The EPM instrument consisted of two open arms (50 × 10 cm) and two closed arms (50 cm × 10 cm × 40 cm) that originated from a central platform (10 × 10 cm). The maze was 50 cm above the ground. This test was performed in a quiet and dark (visibility was 5 m) room. Each rat was placed on the central platform, with their head facing toward one of the open arms. The open arm entry times, close arm entry times and open arm duration were recorded within 5 min after placement, and the percentage of time entering the open arm and the percentage of time staying in the open arm were calculated. The rat was considered to enter a new arm when it introduced four paws in the arm. The EPM test was performed on PND6.

#### 2.4.3. Forced Swimming Test

The forced swimming test (FST) was carried out on PND8 to evaluate the depressive-like behavior of each rat [20]. In the FST, each rat was placed in a water tank (60 × 40 × 50 cm), where the water depth was 30 cm and the water temperature was 24 ± 1 °C. The rat was allowed to adapt to the water for 2 min, and after that, the duration of immobility time of each rat was recorded during the next 4 min. The rats were not allowed to climb the upper edge of the water tank or to stand on the bottom of the tank.

#### 2.4.4. Sucrose Preference Test

The sucrose preference test (SPT), which is a method for detecting anhedonia in animals based on their preference for sweet taste, was performed on PND21. Anhedonia refers to a decline in the ability to experience pleasure, which is an important feature of depression [21]. Each rat was first reared in a single cage without water for 24 h and then given one bottle of 1% sucrose water and one bottle of purified water at the same time. The consumption of sucrose water and pure water during the next 1 h was recorded for each rat. The sucrose preference (%), which is calculated as sucrose water consumption divided by the sum of sucrose water and pure water consumption, was calculated.

**Figure 2:**
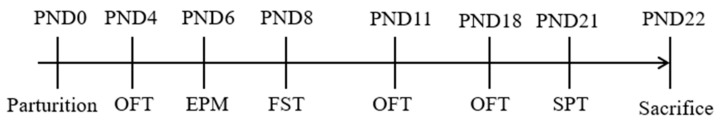
Schedule of behavioral tests. OFT, open field test; EPM, elevated plus maze test; FST, forced swim test; SPT, sucrose preference test.

The schedule of the behavioral tests in the present study is shown in Figure 2.

### 2.5. Sample Collection

The body weight and blood glucose were monitored regularly during lactation, and abnormal conditions such as rejection of lactation, lethargy, immobility and vaginal bleeding were observed and recorded. A blood sample was collected from the orbital venous plexus of each rat on PND7 and PND14. After the behavior test on PND21, each rat was fasted for 12 h and then sacrificed. Blood samples were collected from the orbital venous plexus and serum were separated by 1000× *g* for 15 min. The serum was stored at −80 °C for further analysis. The hippocampus and prefrontal cortex (prefrontal cortex) of the brain were collected and excised on ice. The samples of brain tissue were stored at −80 °C in sterile enzyme-free cryotubes. The right prefrontal cortex and hippocampus of each animal were fixed with 4% paraformaldehyde for immunohistochemical staining. The distal 5 cm colon was dissected rapidly, and the colon contents were collected into a sterile EP tube in a sterile environment. The colon and samples of contents were snap-frozen with liquid nitrogen and stored at −80 °C for future use.

### 2.6. ELISA Quantification of Neurotransmitter

Enzyme-linked immunosorbent assay (ELISA) was used to detect hormone and tryptophan related products in serum, the hippocampus and the prefrontal cortex. The hippocampus and prefrontal cortex were exposed to 9 times the volume of PBS (pH = 7.4), the homogenate was 5000× *g*, at 4 °C for 5 min. The supernatant was collected for ELISA assay according to the instructions of the Kit (Nanjing Jiancheng Bioengineering Institute Co., Nanjing, Zhejiang, China).

### 2.7. Immunohistochemistry (IHC)

Fixed tissue was cut into chips and rehydrated in a graded series of ethanol. The tissue chip was placed in the citrate buffer (pH = 6.0) at 28 °C for 40 min to repair the antigen, then incubated with 3% H_2_O_2_ at 28 °C for 25 min to inactivate the endogenous peroxidase and washed with PBS (pH = 7.4) 3 times. Then, 3% BSA was added, and the chip was sealed at 28 °C for 30 min. After gently shaking off the solution, chips were incubated overnight at 4 °C with the antibody of Anti-IDO2 (ab288067, Abcam, Shanghai, China) or Anti-TPH2 (A06002-2, BOSTER, Wuhan, Hubei, China). On the next day, the biotin-labeled secondary antibody and horseradish peroxidase (HRP)-conjugated streptavidin were added to the tissue chip and incubated at 28 °C for 50 min. Finally, fresh DAB color-developing solution was added. The chips were scanned by a panoramic scanner PANNORAMIC (3DHISTECH, Budapest, Hungary). The software Aipathwell (Servicebio, Wuhan, Hubei, China) was used to analyze the staining intensity and rate of the positive cells. The positive grade was evaluated first: negative, without coloring, 0 point; weak positive, light yellow, 1 point; medium positive, brownish yellow, 2 points; strong positive, sepia, 3 points. Then, we analyzed and calculated the grade, the measurement area, the positive area, the tissue area in the measurement area, the cumulative optical density (IOD), the mean optical density (MOD) and the positive area density (AD). The histochemistry score (H-score) was used for each slide in order to evaluate the staining intensity. H-score = ∑ (pi × i) = (percentage of weak intensity × 1) + (percentage of moderate intensity × 2) + (percentage of strong intensity × 3) [22].

### 2.8. Western Blot

The proteins of the prefrontal cortex, hippocampus and colon were extracted by RIPA lysis buffer (Sigma, St Louis, MO, USA) and homogenized on ice using a glass grinder. Following 20,000× *g*, 4 °C for 20 min, the protein in the supernatant of concentration was determined by BCA protein assay reagent (Thermo Scientific Rockford, IL, USA). After boiling with the loading buffer, it was separated by electrophoresis and transferred to the polyvinylidene difluoride (PVDF) membranes. The membranes were sealed with 5% non-fat milk in TBS-T solution and placed at room temperature for 4 h. The membranes were incubated overnight at 4 °C with the following primary antibodies: (1) rabbit anti-TPH1 antibody (1:1000, Boster, Wuhan, Hubei, China); (2) rabbit anti-IDO2 antibody (1:2000, Boster, Wuhan, Hubei, China); (3) rabbit anti-TPH2 antibody (1:1000, Boster, Wuhan, Hubei, China). After incubation, the membranes were washed with TBS-T solution 3 times. The membranes were incubated with HRP-labeled secondary antibody (Goat Anti-Rabbit IgG, 1:10,000, ABCAM, Shanghai, China) at room temperature for 4 h and then washed with TBS-T solution 3 times. The bands were visualized with ECL (Millipore Corporation, Billerica, MA, USA). All experiments were repeated at least three times. Image-Pro Plus software (Media Cybernetics, Inc. Rockville, MD, USA) was used to analyze the gray intensity values of bands and normalize the results to the counterparts of β-actin.

### 2.9. Microbiota Analysis by 16S Sequencing

#### 2.9.1. DNA Extraction and PCR Amplification

Microbial DNA was extracted using the QIAamp^®^ FastDNA StoolMini Kit (QIAGEN, Hilden, North Rhine-Westphalia, Germany) according to the manufacturer’s protocol. The V3-V4 region of the bacteria 16S rDNA genes were amplified by PCR (95 °C for 3 min, followed by 30 cycles at 98 °C for 20 s, 58 °C for 15 s, and 72 °C for 20 s and a final extension at 72 °C for 5 min) using barcoded primers 341F (5′-CCTACGGGRSGCAGCAG-3′) and 806R (5′-GGACTACVVGGGTATCTAATC-3′). PCR reactions were performed in a 30 μL mixture containing 15 μL of 2 × KAPA Library Amplification ReadyMix, 1 μL of each primer (10 μM), 50 ng of template DNA and ddH_2_O.

#### 2.9.2. Illumina Sequencing and Data Processing

PCR products were detected by 2% agarose gels and purified by AxyPrep DNA Gel Extraction Kit (Axygen Biosciences, Union City, CA, USA). After that, Thermo NanoDrop 2000 (Thermo Fisher Scientific, Waltham, MA, USA) and 2% agarose gel electrophoresis were used for library quality control. A specific 16S primer was designed to amplify the specific region, and about 425 bp amplified fragment was obtained. The paired-end data of PE250 were obtained by sequencing using the Illumina platform, and a longer sequence was obtained by splicing, which was used for 16S analysis. OTUs (Operational taxonomical Units) were clustered with 97% similarity cutoff using Usearch (version 7.0.1090 http://drive5.com/usearch/, accessed on 15 October 2021); each out represents one species. In order to avoid the analysis bias caused by the different sample sequence data size, if the sequence depth was enough, according to the minimum sequence number matching to the OTUs, random drawing was carried out, and Alpha diversity was analyzed. A representative sequence of the read was extracted from each OTU, which was compared with RDP Classifier (http://rdp.cme.msu.edu/, accessed on 15 October 2021); the species of each OTU was classified, and the species abundance table was obtained for follow-up analysis.

### 2.10. Statistical Analysis

All data were analyzed using SPSS (version 25.0, Armonk, NY, USA). The results were all continuous variables and were expressed by mean ± SEM. When normality and equal variance between sample groups were achieved, one-way ANOVA followed by Fisher’s Least Significant Difference (LSD) was used to compare the differences among groups. If failed, one-way ANOVA followed by Tamhane’s test was performed. *p* < 0.05 was the threshold for statistical significance.

## 3. Results

### 3.1. Fasting Plasma Glucose Monitor

**Figure 3:**
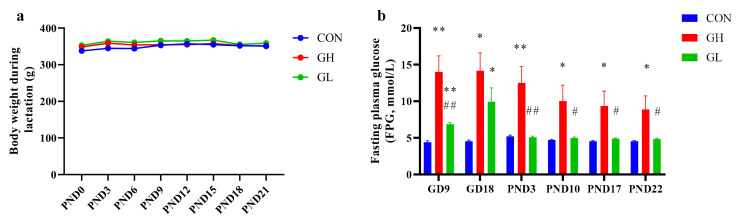
The body weight and FPG during lactation period. (**a**) The maternal body weight during lactation. (**b**) The fasting plasma glucose during pregnancy and lactation. * significant differences vs. CON group, *p* < 0.05. ** significant differences vs. CON group, *p* < 0.01. ^#^ significant differences vs. GH group, *p* < 0.05. ^##^ significant differences vs. GH group, *p* < 0.01.

On PND4, GDM rats with fasting plasma glucose higher than 6.0 mmol/L were put into the GH group, and the other GDM rats were assigned to the GL group. During lactation, the FPG (Figure 3b) of the GH group was significantly higher than that of the CON and GL group. After grouping, the FPG in the gestational period was higher than the CON in the GH group and GL group, and the FPG of the GH group also higher than the GL group. After 8 weeks of HFD, the body weight (Figure 3a) of the CON group was 307.6 ± 4.9 g (*n* = 10), that of the GH group was 327.8 ± 5.7 g (*n* = 12), that of the GL group was 319.0 ± 7.9 g (*n* = 12) and that of the GH group and GL group was increased, but the difference was not statistically significant (*p* = 0.151).

### 3.2. GDM-Induced Postpartum Depression-like Behavior in Rats

**Figure 4:**
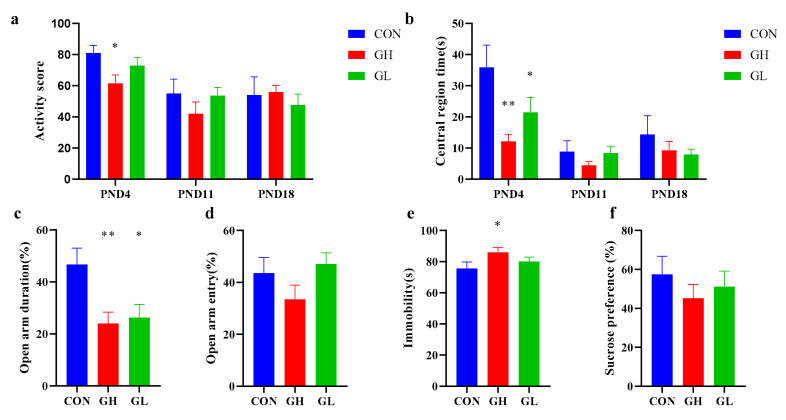
Results of postnatal behavior test in CON, GH and GL group. (**a**) Activity point in the OFT. (**b**) Central region times (s) in the OFT. (**c**) Open arm entry (%) in the EPM in PND6. (**d**) Open arm duration (%) in the EPM test on PND6. (**e**) Immobility times (s) in the FST on PND8. (**f**) Sucrose consumption ratio (%) in the SPT on PND21. * significant differences vs. CON group, *p* < 0.05. ** significant differences vs. CON group, *p* < 0.01.

After parturition, three dams in the GH group were found to refuse to feed their offspring; however this was not the case in the CON and GL groups. In the first OFT at PND4 (Figure 4a), the activity score of the GH group was significantly lower than that in the CON group. No significant difference was found between the GL and CON groups; however, the absolute value of the average score of the GL group was lower than that of CON. The residence time in the central region (Figure 4b) of the GH and GL groups was significantly lower than that of the CON group. 

In the EPM test, comparing with the CON group, the time percentage of staying in the open arm (Figure 4c) was significantly lower in the GH and GL groups. The time of entering the open arm (Figure 4d) in the GH and GL groups was significantly lower than that in CON. It was shown in the FST that that the immobility time of the GH group was significantly lengthened compared with CON; however, no significant difference was found between the GL and CON group (Figure 4e). The SPT showed no significant difference between the groups, although from the bar chart, the sucrose preference of the GH group was slightly lower than the CON group (Figure 4f). 

Based on the above behavioral results, GDM dams were more likely to develop postpartum depression, and those whose blood sugar did not recover after delivery were at greater risk.

### 3.3. HPA Axis Hormone

**Figure 5:**
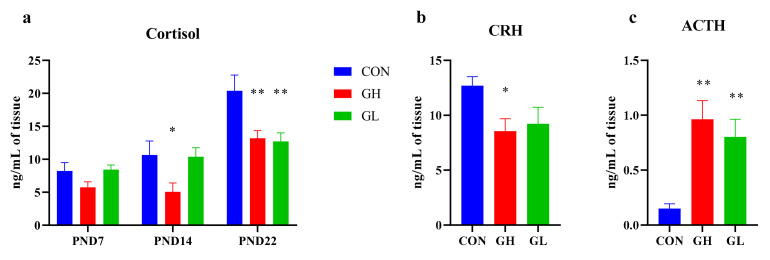
The levels of serum HPA axis hormones. (**a**) The serum COR level during lactation. (**b**) The serum CRH level on PND22. (**c**) The serum ACTH level on PND22. * significant differences vs. CON group, *p* < 0.05. ** significant differences vs. CON group, *p* < 0.01.

As shown in Figure 5, the average level of serum COR in GH group dams was significantly lower than that of the CON group. Interestingly, the level of COR in the GL group was almost the same as that in CON during lactation, but decreased significantly after weaning (*p* = 0.002). Accordingly, the level of adreno-cortico-tropic-hormone (ACTH) was significantly increased, and corticotropin releasing hormone (CRH) was significantly decreased (*p* = 0.037). The changes of ACTH and CRH in the GH group were greater than in the GL group.

### 3.4. Expression of Trp Pathway Neurotransmitters

**Figure 6:**
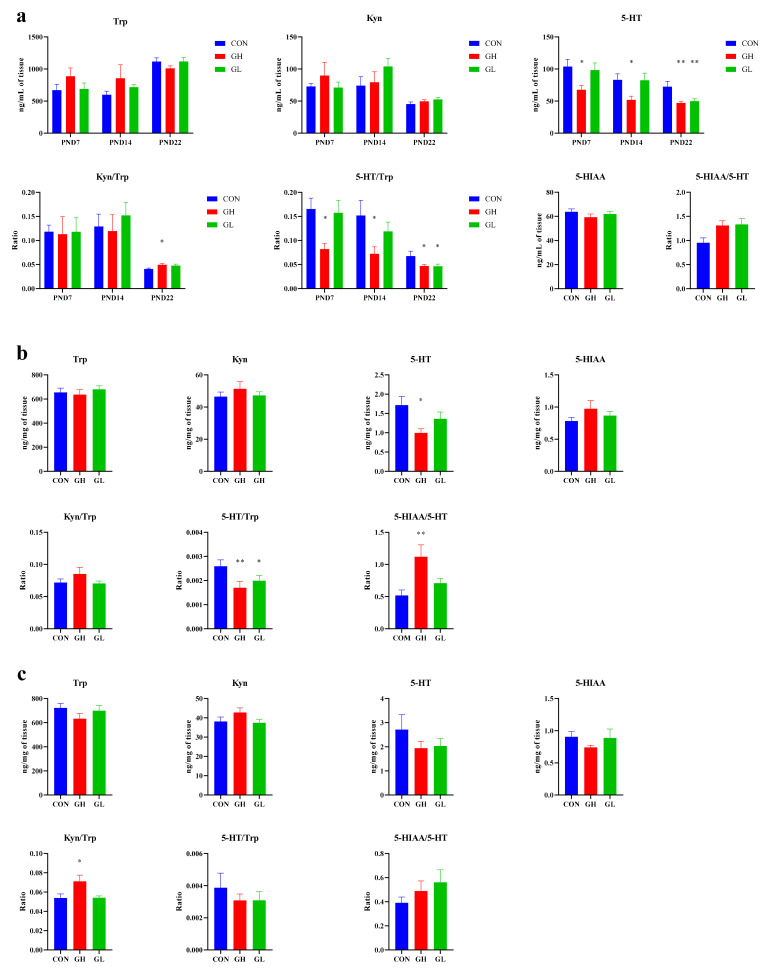
Trp and its metabolites levels in (**a**) serum, (**b**) the hippocampus and (**c**) the prefrontal cortex. * significant differences vs. CON group, *p* < 0.05. ** significant differences vs. CON group, *p* < 0.01.

During the lactation period, the trends of serum Trp and its major metabolites were not significantly different among the groups (Figure 6). The 5-HT level in the GH group was significantly lower than that in CON group; however, no such significance was found between the GL and CON groups. The 5-HT/Trp ratio decreased significantly in the GH group. The content of Kyn, another major metabolite of Trp, was slightly higher in the GH and GL groups than in the CON group, although no statistical difference was found.

Trp and its metabolites in serum, the hippocampus and the prefrontal cortex were measured on PND22. In general, 5-HT concentration and 5-HT/Trp ratio in the GH and GL groups were lower than those in the CON group, and the ratios of Kyn, Kyn/Trp and 5-HIAA/5-HT were higher. In the serum (Figure 6a), the concentration of 5-HT and the ratio of 5-HT/Trp in the GH and GL groups were significantly lower than those in the CON group, and the ratio of 5-HIAA/5-HT was significantly higher than that in the CON group. The Kyn and Trp ratios in the GH group were significantly higher than those in the CON group. In the hippocampus (Figure 6b), the 5-HT and 5-HT/Trp in the GH group was significantly lower than that in CON, the ratio of 5-HIAA/5-HT was significantly higher than that in the CON group, and the ratio of 5-HT/Trp in the GL group was significantly lower than that in the CON group, the differences were statistically significant. In the prefrontal cortex (Figure 6c), the ratio of Kyn/Trp in the GH group was significantly higher than in the CON group. The increase in Kyn/Trp indicated the trend that Trp metabolism was changed from the Trp-5-HT pathway to the Trp-Kyn pathway. Compared with the GH group, the level of Kyn and the ratio of Kyn/Trp in the GL group increased to some extent, and the ratio of 5-HT/Trp also increased, which indicated that the Trp-5-HT metabolic pathway was improved. 

### 3.5. Expression of Key Enzymes in Trp Metabolic Pathway in the Brain and Colon

**Figure 7:**
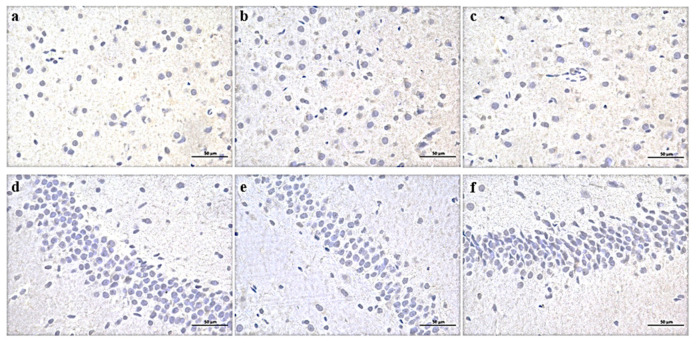
Expression of IDO in prefrontal cortex (**a**–**c**) and hippocampus (**d**–**f**) of three groups of rats. (**a**,**d**) CON group, (**b**,**e**) GH group, (**c**,**f**) GL group. DAB × 400.

**Figure 8:**
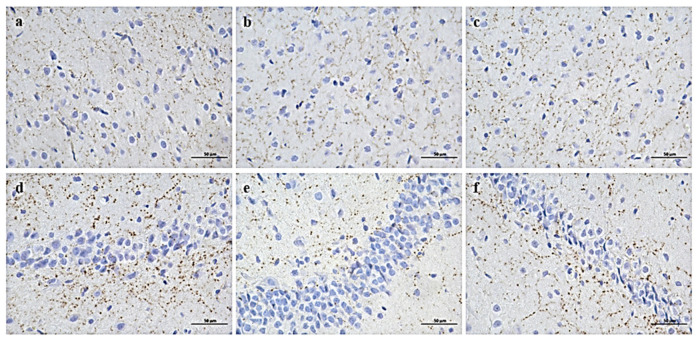
Expression of TPH2 in prefrontal cortex (**a**–**c**) and hippocampus (**d**–**f**) of three groups of rats. (**a**,**d**) CON group, (**b**,**e**) GH group, (**c**,**f**) GL group. DAB × 400.

The expression of IDO in the prefrontal cortex and hippocampus of the GH group was significantly increased, while the expression of TPH2 was decreased. The H-scores of the prefrontal cortex and hippocampus chips of the GH group were significantly increased compared to CON (Table 1, Figure 7). There was no significant difference in the intensity of positive staining among groups, but the positive area of the GH group was the largest. Similarly, a trend was found in TPH2 expression both in the prefrontal cortex and in the hippocampus of the GH group; however, no statistical difference was found in the hippocampus (Table 1, Figure 8). The IDO expression of the GL group was slightly higher than that of CON in the prefrontal cortex (*p* = 0.061) and parallel the level of the CON group in the hippocampus.

**Figure 9:**
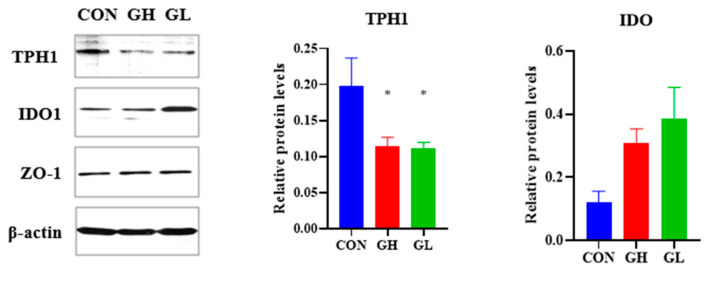
Western blot of rate-limiting enzymes that induced Trp metabolism in colon. * significant differences vs. CON group, *p* < 0.05.

Western blotting of the colon showed that the expression of TPH1 was significantly decreased (*p* = 0.031) and IDO was increased in the GH group (*p* = 0.066) (Figure 9). The expression of IDO in the GL group was significantly increased (*p* = 0.004), while the expression of TPH1 was decreased (*p* = 0.064). The above results revealed that GDM affected the expression of key enzymes in the Trp pathway in the colon, prefrontal cortex and hippocampus, TPH changed significantly in the colon and prefrontal cortex, and IDO changed significantly in the colon, prefrontal cortex and hippocampus. Additionally, compared with the GH group, the GL group was improved.

### 3.6. Gut Microbiota Changes

**Figure 10:**
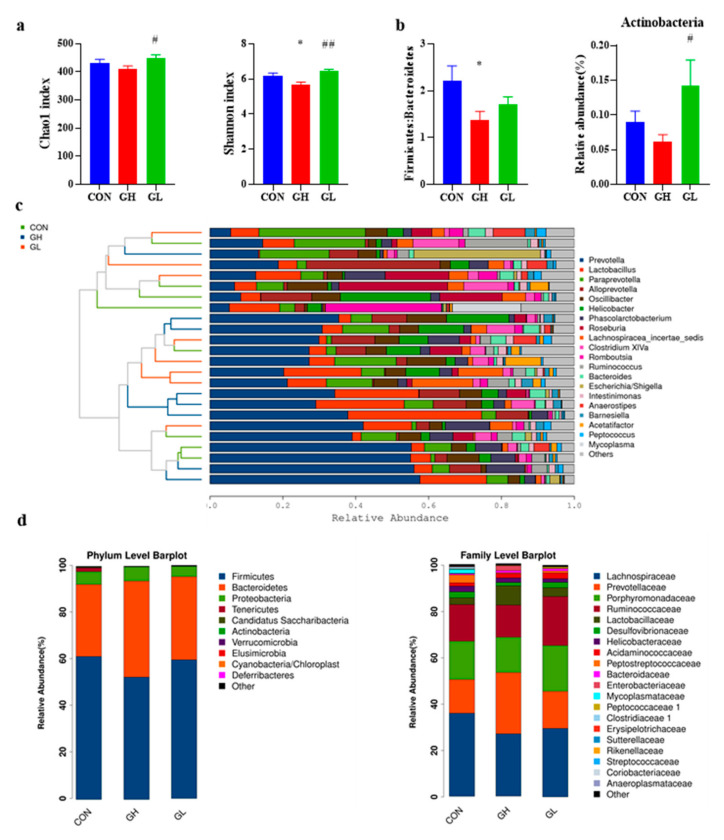
Species diversity and composition of gut microbiota. (**a**) Chao1 and Shannon index describing microbial α diversity. (**b**) *Firmicutes*:*Bacteroidetes* ratio and the relative abundance of *Actinobacteria*. (**c**) Cluster analysis of species at genus level. (**d**) Bar chart of relative abundance of species at phylum and family levels. * significant differences vs. CON group, *p* < 0.05. ^#^ significant differences vs. GH group, *p* < 0.05. ^##^ significant differences vs. GH group, *p* < 0.01.

The Chao1 index and Shannon index (Figure 10a) of the GH group decreased significantly, which indicated that the diversity of gut microbiota in the GH group decreased. However, no such changes found in the GL group. Through the species cluster analysis (Figure 10c), the composition of the CON and GL groups is roughly divided into one cluster and the GH group itself is another cluster, indicating that the GH group and the other two groups had significant differences in the composition of gut microbiota.

**Figure 11:**
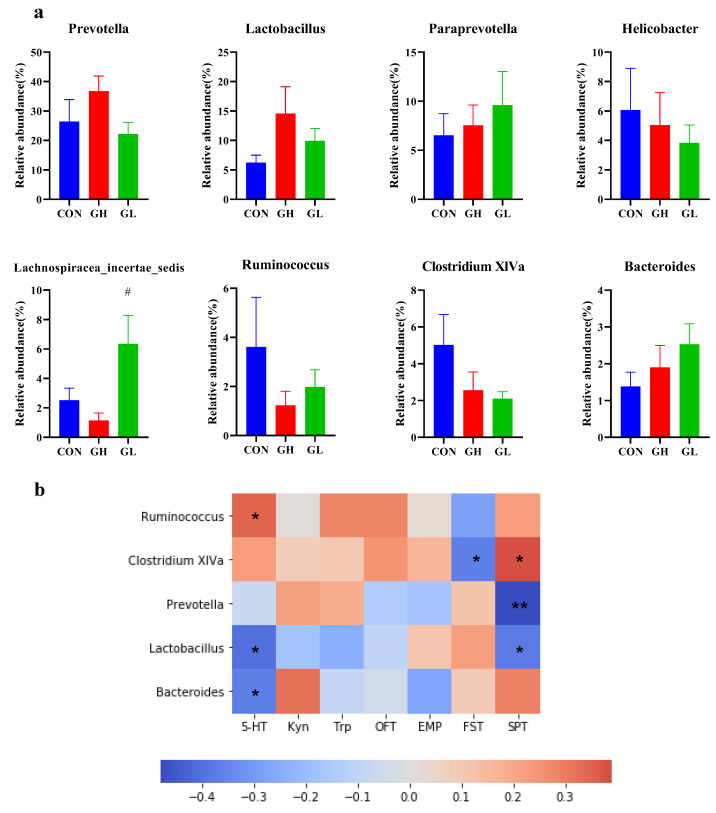
Difference of relative abundance of gut microbiota at the genus level. (**a**) The relative abundance of obviously changed genera. (**b**) The Pearson correlation heatmap focusing on the correlations between microbiota and serum Trp metabolic pathway markers, behavioral test. Scale indicates the level of positive (red) or negative (blue) correlation. * *p* < 0.05, ** *p* < 0.01. ^#^ significant differences vs. GH group, *p* < 0.05.

Taxonomic shifts were also investigated, and the ratio of gut microbiota varied among the different groups at the phylum level (Figure 10d). The most common were *Firmicutes*, *Bacteroidetes* and *Proteobacteria*. The ratio of *Firmicutes*/*Bacteroidetes* (Figure 10b) in the GH and GL groups increased, and the changes in the GH group were statistically significant (*p* = 0.015). In addition, the relative abundance of *Actinobacteria* decreased in the GH group and increased in the GL group (*p* = 0.025) compared with CON. GDM increased the *Firmicutes*/*Bacteroidales* ratio, due to an underlying change in the abundance of the orders *Clostridiales* and *Bacteroidales*. The decrease of *Clostridiales* in the GH and GL groups (*p* = 0.033) and the increase of *Bacteroidales* in the GH group (*p* = 0.021) were both statistically significant. The family-level results (Figure 10d) showed that the relative abundance of *Prevotellaceae* in the GH group was significantly higher than that of the CON group (*p* = 0.018). The relative abundance of *Ruminococccaceae*, *Peptococciaceae* 1 and *Coriobacteriaceae* in the GL group was significantly higher than that of the CON group. In addition, the highest relative abundance, *Lachnospirnaceae*, was slightly decreased in the GH and GL groups. At the genus level (Figure 11), the relative abundance of typical microorganisms, *Prevotella*, *Lactobacillus* and *Paraprevotella*, increased in the GH group, with *Helicobacter*, *Clostridium* XlVa and *Ruminococcus* decreased.

In order to further elucidate the potential relationship between Trp metabolic and gut microbiota, the correlation between serum markers, behavioral results and microbial relative abundance was analyzed. There was a significant positive correlation between *Ruminococcaceae* and 5-HT in serum, and a significant negative correlation between *Lactobacillus*, *Bacterioides* and 5-HT. In addition, there was a strong positive correlation between *Bacteroides* and Kyn level, although it was not statistically significant. *Clostridium* XlVa showed a significant positive correlation with behavioral outcomes and improved FST and SPT performance. *Prevotella* and *Lactobacillus* showed a negative relationship with the performance in behavior tests.

## 4. Discussion

The present study indicated that GDM could directly induce postpartum depression-like behavior in rats. It was observed that 3 out of 12 dams in the GH group refused to feed their offspring, which is a typical symptom and one of the most serious clinical manifestations of postpartum depression [23]. This was also indicated by subsequent behavioral tests, in which animals in both the GH and GL groups exhibited reduced voluntary and exploratory activity in the OFT and EMP tests in the early postpartum period. This result not only demonstrated that GDM can increase the risk of postpartum depression-like behavior, but also suggested that the degree of postpartum depression may be related to plasma glucose level. The higher the plasma glucose level during pregnancy, the more likely it will not recover to normal in the short term, and the greater the risk of postpartum depression-like behavior. Even if the plasma glucose recovered after delivery, postpartum depression-like behavior still occurred in the GL group. Although the results were found in rats, it is likely that humans may have a similar condition, and attention should be paid to the mental health of recovered GDM patients.

In this study, GDM rats were fed with basic chow rather than HFD after parturition. Blood glucose levels of GDM rats were differentiated; some of them recovered to normal (GL group), and others did not (GH group). Through the retrospective study, we found that the blood glucose of the GH group was high during pregnancy, making recovery difficult. The GH and GL groups represent two types of GDM patients with different clinical outcomes. Therefore, the findings may have more representative and practical significance.

As a classical theory of depression, the hyperactivity of the HPA axis, especially the increase of cortisol, is one important mechanism leading to depression [6]. The constant increase of cortisol caused by long-term stress can damage hippocampal neurons [24]. In the classic animal model of depression, the chronic unpredictable stress model (CUMS), an increase of cortisol level indicates the successful establishment of the animal depression model [25]. Continuous injection of high levels of cortisol after delivery (COR-based model) is also one method to establish the PPD animal model [26]. Interestingly, in this study, the plasma cortisol level did not increase in the GDM group. On the contrary, the plasma cortisol levels decreased in both the GH and GL groups. This may be due to the high blood glucose level of GDM dams, which through negative feedback regulation, inhibited the secretion of cortisol, resulting in decreased cortisol level. It also may be related to the changes in cortisol clearance rate and metabolism. The decrease of cortisol level was mediated by a negative HPA axis feedback pathway, leading to an increase in ACTH secretion and a decrease in CRH secretion. This suggests that elevated cortisol level is not a necessary condition for postpartum depression. In combination with the other result of this study, we can speculate that hyperglycemia, a consequence of elevated cortisol, may be a physiological factor in postpartum depression-like behavior.

5-HT, one important metabolite of tryptophan, is a neurotransmitter that can induce happy mood. 5-HT is also involved in the process of neuron production and neural circuit formation. In a study involving TPH2 knock-out mice, lack of 5-HT affected the normal connections of neurons in the brain, leading to neurodevelopmental disorders [27]. Other studies have shown that postpartum depression patients have significantly lower levels of 5-HT, and this can lead to mood and behavior control disorders [28,29]. Abnormal metabolism of 5-HT is one of the classic mechanisms of depression, and the antidepressant fluoxetine plays its role by raising 5-HT levels in the brain. In this study, it was found that the serum 5-HT level of GDM dams decreased significantly during the entire lactation period. The difference of 5-HT level between the GH and GL groups was consistent with the results of the behavioral experiment, which further illustrates the effect of blood glucose level on postpartum depression-like behavior.

Moving the target from 5-HT to the whole Trp metabolic pathway containing 5-HT, we found that not just 5-HT, but other important metabolites in the Trp pathway that produce 5-HT, also differed between GDM dams and normal dams. The levels of Kyn, another important metabolite of Trp, increased in serum and the brain. Kyn, as the most important metabolite of Trp, is produced in the reaction catalyzed by IDO and eventually metabolized into neurotoxic quinolinic acid and neuroprotective kynurenic acid. The role of Kyn in the pathogenesis of depression has also been confirmed by a number of studies [30]. Some population studies have pointed out that the plasma level of Trp in patients with major depression is decreased, and the Kyn/Trp ratio is increased and associated with suicidal ideation [31]. Animal studies have also shown that chronic mild stress in mice leads to increased activity of the Kyn pathway; depression-like behavior induced by chronic stress can be improved by using IDO inhibitor 1-methyl-D-tryptophan (1-MDT) to inhibit the decrease of IDO activity [32]. In addition, the downstream metabolite of Kyn, 3-hydroxykynurenine (3-HK), also induced depressive-like behavior in mice [33]. These results were consistent with the present study. The migration of the Trp pathway from 5-HT to Kyn may be one of the mechanisms inducing depressive-like behavior in postpartum mothers with GDM.

By measuring the key rate-limiting enzymes of the Trp pathway, it was found that the shift of Trp metabolism to Kyn in serum, the hippocampus and the prefrontal cortex was due to the decrease of the expression of TPH1 and TPH2 and the up-regulation of the expression of IDO. Trp produces 5-HT with the tryptophan hydroxylase, which is divided into two subtypes: TPH1 in enterochromaffin cells (ECs) and TPH2 in 5-HT neurons in the brain. 5-HT is mainly produced by ECs, so the decrease of TPH1 expression in the colon of GDM rats leads to the decrease of 5-HT content in serum, and the accumulation of Trp is caused by the disruption of synthesis. The production of Kyn is dependent on indoleamine 2,3-dioxygenase, which is expressed in many tissues and cells. The up-regulation of IDO in the colon, prefrontal cortex and hippocampus resulted in the increase of Kyn and Kyn/Trp ratios in serum and the brain tissue of GDM rats. From the above results, it can be concluded that the physiological basis of postpartum depression-like behavior induced by GDM is closely related to the metabolic pathway of Trp.

The brain–gut axis serves as a bidirectional connection between the central nervous system and the digestive tract, where the gut microbiota plays a major role. Trp is metabolized in the gut through three main pathways: (1) the microorganisms metabolize Trp directly into tryptamine, 3-Methylindole, indole propionic acid, etc.; (2) the 5-HT pathway involved in TPH1 in ECs; (3) the Kyn pathway involved in IDO in colon [34]. Because Trp and Kyn can directly penetrate the blood–brain barrier (BBB), the effect of gut microbiota on the Trp metabolism pathway can further regulate the function of the central nervous system [35].

Although the mechanism of the effect of gut microbiota on IDO is not clear, it has been proven that the expression of IDO in the intestinal tract of germ-free (GF) mice is deficient compared with that of normal mice [36]. Gut microbiota is involved in regulating the immune response and the expression of inflammatory factors, such as IFN-γ, which depend on gut microbiota. In agreement with Eva [37], our study demonstrated that GDM can induce an inflammatory state, thereby increasing immune factors involved in the overexpression of IDO. In some animal experiments, mice were successfully induced to exhibit depressive-like behavior by injection of lipopolysaccharide (LPS) to stimulate the immune response [33]. Both the previous study and this study have confirmed that GDM can put the body in an inflammatory state, so the increase of immune factors induces the overexpression of IDO.

With the development of brain–gut axis theory, many researchers have shown the relationship between gut microbiota, diabetes and depression. The dysbacteriosis caused by GDM was not only reflected in the decrease of species diversity, but also was similar to the dysbacteriosis of postpartum depression. There were also significant differences in gut microbiota between patients with postpartum depression and healthy controls, excluding the effects of other diseases [10], with increased abundance of postpartum depression and decreased abundance of *Firmicutes* in patients with postpartum depression. This was consistent with the results of dysbacteriosis caused by GDM, which may be caused by the change of GDM microflora, which can affect the metabolism of 5-HT and Kyn.

This study found a strong positive correlation between *Bacteroides* and Kyn levels. Although the exact mechanism is unknown, other studies have shown that the increase in the relative abundance of *Bacteroides* is associated with an increased Kyn/Trp ratio in serum and the hippocampus in the maternal separation rat model [38]. It is suggested that the gut microbiota may affect Kyn metabolism through inflammatory reaction, which was accompanied by elevated proinflammatory cytokine levels in the colon (IFN-γ and IL-6) and serum (IL-1β). It has been proven that the expression of IDO in the intestinal tract of GF mice is lower than that of normal mice, suggesting that the effect of gut microbiota on metabolism of Kyn may be realized by regulating the expression of IDO [36].

Similarly, *Ruminococcu* and *Clostridium*, which had a positive effect on 5-HT in this study, have been shown to be associated with 5-HT metabolism in other studies. It has been found that the relative abundance of *Clostridium* is closely related to the concentration of 5-HT in the colon [39]. *Ruminococcu*, similar to *Clostridium*, is widely distributed in the human body, and the relative abundance of 5-HT is decreased in GDM rats. Trp levels in the prefrontal cortex were positively correlated in another study [40]. Compared with SPF mice, the ECs of GF mice were larger and more functional and could express more TPH1, suggesting that the gut microbiota might affect the 5-HT pathway through TPH [41]. *Lactobacillus*, which negatively affects behavioral outcomes in this study, has been shown in other studies to reduce 5-HT levels in the brain and hippocampus [42]. Therefore, the effect of gut microbiota on 5-HT metabolism cannot be ignored.

**Figure 12:**
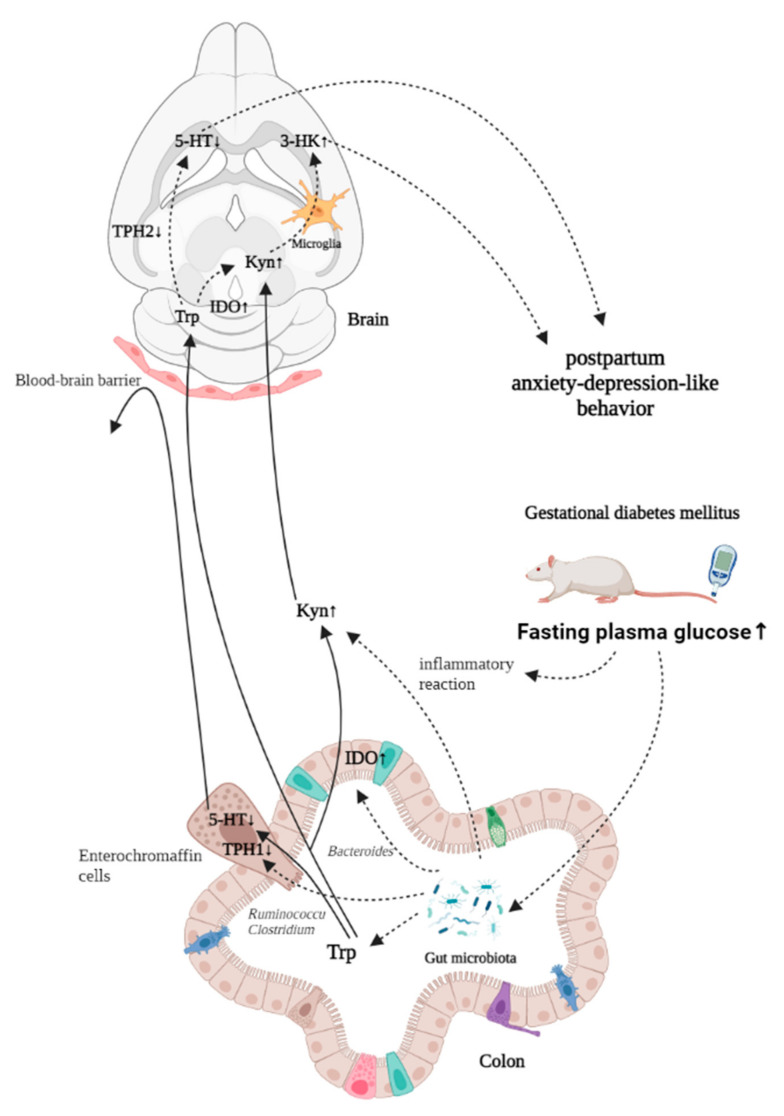
The microbial-gut-brain axis model of postpartum depression-like behavior induced by GDM.

With the series of changes caused by GDM, it is reasonable to speculate that GDM affects the Trp metabolic pathway by changing the gut microbiota, thus changing the behavior of the animal. The mechanism summarized in this study is shown in Figure 12.

## 5. Conclusions

GDM rats showed postpartum depression-like behavior. The physiological basis of this phenomenon is related to the abnormality of the Trp metabolic pathway, especially the decrease of 5-HT levels. GDM may affect the metabolism of Trp by changing the composition of gut microbiota; in particular, the specific microbiota may affect the metabolism of Kyn and 5-HT, which influence the metabolic state of central nervous system. The inflammatory response to GDM also plays a synergistic role. 

The change of the HPA axis hormone is not consistent with other depression models. However, whether it plays a positive role in postpartum depression in light of the effects of GDM needs to be further discussed. In this study, a GDM animal model was used to study postpartum depression, which provides a novel concept for the pathogenesis of postpartum depression, as well as a potential biological and a bacterial target for the prevention and treatment of postpartum depression.

## Figures and Tables

**Table 1:** Comparison of IDO and TPH2 immuno-positive MOD, AD and H-score in different brain regions of rats in each group.

Tissue-Protein	IHC	CON	GH Group	GL Group
prefrontal cortex-IDO	MOD	0.161 ± 0.001	0.158 ± 0.001	0.159 ± 0.001
	AD	0.017 ± 0.002	0.038 ± 0.004 **	0.027 ± 0.004 ^#^
	H-score	10.737 ± 1.024	24.495 ± 2.834 **	17.136 ± 2.690 ^#^
hippocampus-IDO	MOD	0.156 ± 0.002	0.160 ± 0.001	0.155 ± 0.001
	AD	0.020 ± 0.004	0.028 ± 0.003 *	0.019 ± 0.002 ^#^
	H-score	12.569 ± 2.104	17.725 ± 1.991 *	12.247 ± 1.176 ^#^
prefrontal cortex-TPH2	MOD	0.233 ± 0.003	0.235 ± 0.006	0.237 ± 0.005
	AD	0.005 ± 0.000	0.004 ± 0.000 **	0.004 ± 0.000
	H-score	3.089 ± 0.161	2.342 ± 0.114 **	2.644 ± 0.237
hippocampus-TPH2	MOD	0.259 ± 0.002	0.262 ± 0.004	0.263 ± 0.003
	AD	0.006 ± 0.001	0.005 ± 0.001	0.006 ± 0.000
	H-score	3.990 ± 0.413	3.407 ± 0.418	3.741 ± 0.433

* *p* < 0.05 and ** *p* < 0.01 indicate significant differences vs. CON group. ^#^ significant differences vs. GH group, *p* < 0.05.

## Data Availability

The datasets used during the current study are available from the corresponding author on reasonable request.
